# A Prospective, Cohort Study of SITOIGANAP to Treat Glioblastoma When Given in Combination With Granulocyte-Macrophage Colony-Stimulating Factor/Cyclophosphamide/Bevacizumab/Nivolumab or Granulocyte-Macrophage Colony-Stimulating Factor/Cyclophosphamide/Bevacizumab/Pembrolizumab in Patients Who Failed Prior Treatment With Surgical Resection, Radiation, and Temozolomide

**DOI:** 10.3389/fonc.2022.934638

**Published:** 2022-06-28

**Authors:** Daniela A. Bota, Thomas H. Taylor, Naomi Lomeli, Xiao-Tang Kong, Beverly D. Fu, Axel H. Schönthal, Samuel Singer, Deborah T. Blumenthal, Frank M. Senecal, Helena Linardou, Evangelos Rokas, Dimitris G. Antoniou, Virgil E. J. C. Schijns, Thomas C. Chen, Joseph Elliot, Apostolos Stathopoulos

**Affiliations:** ^1^ Department of Neurology, University of California Irvine, Irvine, CA, United States; ^2^ Department of Neurological Surgery, University of California Irvine, Irvine, CA, United States; ^3^ Chao Family Comprehensive Cancer Center, University of California Irvine, Irvine, CA, United States; ^4^ Department of Epidemiology and Biostatistics, University of California Irvine, Irvine, CA, United States; ^5^ Department of Molecular Microbiology and Immunology, Keck School of Medicine, University of Southern California, Los Angeles, CA, United States; ^6^ John Theurer Cancer Center, Hackensack University Medical Center, Hackensack, NJ, United States; ^7^ Neuro-oncology Division, Tel-Aviv Sourasky Medical Center, Tel-Aviv University, Tel-Aviv, Israel; ^8^ Department of Hematology and Oncology, Northwest Medical Specialties, Tacoma, WA, United States; ^9^ Fourth Oncology Department and Comprehensive Clinical Trials Center, Metropolitan Hospital, Athens, Greece; ^10^ Department of Neurosurgery, Henry Dunant Hospital Center, Athens, Greece; ^11^ Epitopoietic Research Corporation (ERC), Gembloux, Belgium; ^12^ Epitopoietic Research Corporation (ERC), Pasadena, CA, United States; ^13^ Department of Neurosurgery, Keck School of Medicine, University of Southern California, Los Angeles, CA, United States; ^14^ Department of Pathology, Keck School of Medicine, University of Southern California, Los Angeles, CA, United States

**Keywords:** recurrent glioblastoma, SITOIGANAP, ERC1671, immunotherapy, GBM vaccine

## Abstract

**Background:**

Glioblastoma (GBM) is the most common primary, malignant brain tumor in adults and has a poor prognosis. The median progression-free survival (mPFS) of newly diagnosed GBM is approximately 6 months. The recurrence rate approaches 100%, and the case-fatality ratio approaches one. Half the patients die within 8 months of recurrence, and 5-year survival is less than 10%. Advances in treatment options are urgently needed. We report on the efficacy and safety of a therapeutic vaccine (SITOIGANAP: Epitopoietic Research Corporation) administered to 21 patients with recurrent GBM (rGBM) under a Right-to-Try/Expanded Access program. SITOIGANAP is composed of both autologous and allogeneic tumor cells and lysates.

**Methods:**

Twenty-one patients with rGBM received SITOIGANAP on 28-day cycles in combination with granulocyte-macrophage colony-stimulating factor (GM-CSF), cyclophosphamide, bevacizumab, and an anti-programmed cell death protein-1 (anti-PD-1) monoclonal antibody (either nivolumab or pembrolizumab).

**Results:**

The mPFS was 9.14 months, and the median overall survival (mOS) was 19.63 months from protocol entry. Currently, 14 patients (67%) are at least 6 months past their first SITOIGANAP cycle; 10 patients (48%) have received at least six cycles and have a mOS of 30.64 months and 1-year survival of 90%. The enrollment and end-of-study CD3^+^/CD4^+^ T-lymphocyte counts strongly correlate with OS.

**Conclusions:**

The addition of SITOIGANAP/GM-CSF/cyclophosphamide to bevacizumab and an anti-PD-1 monoclonal antibody resulted in a significant survival benefit compared to historic control values in rGBM with minimal toxicity compared to current therapy.

## Introduction

Glioblastoma (GBM) is the most common and aggressive of all malignant central nervous system (CNS) brain tumors in adults and has a poor prognosis. With an incidence of 3.2 per 100,000, GBM is rare compared with many non-neurologic malignancies, but inevitably fatal. With the current standard of care, the 5-year survival for GBM is only 6.8% (1). The addition of Optune^®^ (tumor treating fields) to the standard of care treatment for primary GBM, surgical resection followed by radiation therapy (RT) and temozolomide (TMZ), yields a median progression-free survival (mPFS) of 6.7 months and median overall survival (mOS) of 20.9 months (2). Effective treatment options for recurrent GBM (rGBM) are limited, yielding estimates of mOS between 5 and 11 months post-recurrence (3). Second-line therapy options for rGBM include nitrosoureas, TMZ rechallenge, bevacizumab, tumor treating fields, and surgical resection but are not curative. Optune^®^, the latest Food and Drug Administration (FDA)-approved therapy for GBM, yields a mOS of 6.6 months in rGBM, but its cumbersome nature and demanding regimen seem to limit its use (4, 5). Bevacizumab (Avastin), a humanized anti-VEGF monoclonal antibody, was approved for rGBM in the United States in 2009, based on two phase II trials demonstrating 6-month PFS rates of 29% and 43% (6, 7). Once the tumor progresses on bevacizumab, further interventions are limited. Re-irradiation may be considered but is not an option for all cases. Novel therapies for GBM, and especially rGBM, are urgently needed (8).

Immunotherapies can improve the prognosis in noted cases of advanced and metastatic oncologic conditions and may have potential as a therapeutic strategy for GBM. Epitopoietic Research Corporation (ERC) has developed a vaccine-based immunomodulatory therapy aimed at GBM, designated as “SITOIGANAP” (ERC1671/Gliovac™). SITOIGANAP is composed of allogeneic and autologous primary irradiated/inactivated whole tumor cells and lysates (detailed below), administered in combination with oral cyclophosphamide (CYP) and granulocyte-macrophage colony-stimulating factor (GM-CSF) to support immune system priming (9, 10). ERC demonstrated the effectiveness of inactivated tumor cell and tumor-cell lysate vaccination in preclinical models (11, 12), and early-stage clinical trials have shown promise (9, 10, 13, 14). In 2018, interim results from a phase II, double-blind, randomized, placebo-controlled clinical trial (NCT01903330) of SITOIGANAP in bevacizumab-naïve patients with rGBM yielded a mOS of 12 months in patients treated with SITOIGANAP plus bevacizumab, compared to 7.5 months in the placebo-plus-bevacizumab group (9). Additionally, that study suggested that CD3^+^/CD4^+^ T-lymphocyte count from peripheral blood is a marker for OS in patients treated with SITOIGANAP.

The results of a cohort of nine rGBM patients treated with SITOIGANAP under a compassionate-use exemption protocol showed a 6-month OS of 100%, 12-month OS of 40%, and mOS of 10.5 months, compared to a 6-month OS of 33% and mOS of 5.3 months in historic controls (10). Reported adverse events (AEs) in these patients have been mild, mostly injection site reactions. These results suggest that SITOIGANAP is well-tolerated and may increase OS in rGBM patients compared to the current standard of care.

Therapeutic inhibition of immune checkpoint pathways, such as the programmed cell death protein-1 (PD-1)/programmed death ligand-1 (PD-L1), has been explored in randomized clinical trials for newly diagnosed GBM (nGBM) and rGBM. Two recently published studies report encouraging results of neoadjuvant anti-PD-1 therapy in patients with rGBM prior to debulking surgery: Cloughesy et al. (15) administered a single dose of pembrolizumab to 16 participants prior to tumor resection, while 16 control participants underwent tumor resection without preoperative pembrolizumab. Neoadjuvant pembrolizumab was associated with a significant OS benefit (13.7 vs. 7.5 months), a marked shift in the tumor gene expression profile, an expansion of systemic TCR clones, and distinct changes in myeloid and lymphoid populations within the tumor and periphery. Schalper et al. (16) conducted a single-arm phase II study in which 29 participants (both treatment-naïve GBM and rGBM) received one dose of nivolumab before tumor resection and continued to receive nivolumab postoperatively. No apparent OS benefit was seen for rGBM, but 2/3 of nGBM patients survived >28 months. Together, these findings suggest that neoadjuvant PD-1 blockade could effectively enhance local and systemic antitumor responses and have fueled interest in combining neoadjuvant PD-1 blockade with additional immunomodulatory agents. This is the first publication to report the findings of combining SITOIGANAP with anti-PD-1 therapy in rGBM patients.

## Methods

### Study Design

This was a prospective, cohort study of 21 rGBM patients treated with SITOIGANAP on a Right-to-Try (RTT; in the USA) or compassionate use (outside the USA) basis. All 21 participants received adjuvant anti-PD-1 therapy, but by physician’s choice, nine also received neoadjuvant anti-PD-1 therapy. The primary endpoints analyzed were PFS and OS, dating from the surgery when tumor tissue was harvested to produce the vaccine. Counts of CD3^+^/CD4^+^ T-lymphocytes from peripheral blood were secondary endpoints. Results are presented for the entire cohort and stratified by course of anti-PD-1 treatment, MGMT status of the tumor, and the number of cycles of SITOIGANAP received.

### Vaccine Composition and Production

SITOIGANAP (ERC1671/Gliovac™) is composed of primary irradiated/inactivated whole tumor cells and lysates from autologous and allogeneic tissue. The autologous tissue is harvested from a debulking surgery: the allogeneic tissue is from three anonymous GBM patients and acquired from a tissue bank (Tissue Bank of the Netherlands). Donors are matched with the recipient on Class 1 and Class 2 HLA molecular markers. SITOIGANAP is manufactured under good manufacturing practice (GMP) conditions. The GBM tissues collected during clinically indicated resection surgeries are handled, transported, received, and released by a bank of human tissue. Before release, the tissue is tested for the absence of transmissible infections, including HIV, HBV, HCV, CMV, HTLV, and *Treponema pallidum*/syphilis. Samples are sent under temperature-controlled conditions to the SITOIGANAP manufacturing site, where cells are isolated by mechanical dissection. Isolated cells are counted and haptenized with 1-fluoro-2,4-dinitrofluorobenzene to improve immunogenicity. Haptenized cells are divided into two parts: one is preserved for freezing in a sucrose medium, and the other is lysed by osmotic shock. Both parts are further irradiated with 25-Gy gamma radiation to inactivate replication competence and ensure sterility. All preparations are stored at −80°C (9, 10, 13).

### Regulatory Pathway and Ethics

Prior to the debulking surgery, all participants provided written, informed consent to bank the surgical specimen, which was used to manufacture their doses of SITOIGANAP and used as an allogeneic component of the vaccine for other patients. Patients indicated whether their consent would remain in force if they, for any reason, withdrew from the program. Separate, written, informed consent to be treated with SITOIGANAP was obtained prior to administration of the therapy. Tissue was resected, transported, and handled in accordance with international tissue banking standards and compliance with the protocols of the Tissue Bank of the Netherlands.

Most patients were treated in the United States under a RTT program operated in compliance with the Federal RTT Act passed into law in May 2018 and State RTT laws when applicable. ERC chose to deliver an RTT program to patients to obtain indemnification protection offered by the RTT law, limit risk to traditional drug development specific to FDA use of AE data in a fragile patient population, and provide treatment plan flexibility for eligible physicians and patients. All cases received prospective Institutional Review Board (IRB) review and approval. Annual reports were delivered to the Department of Health and Human Services (DHHS) as required by law. Patients receiving early access to SITOIGANAP in other countries (Israel and Thailand) did so under regulatory pathways available in those jurisdictions. As with the patients in the United States, all cases received the dual protection of prospective IRB approval and written informed consent.

### Patient Characteristics

Patients eligible to receive SITOIGANAP *via* the ERC RTT/Expanded Access Program must have 1) histologically confirmed WHO grade IV malignant glioma, 2) documented failure of first-line, standard of care therapy, and 3) Karnofsky performance score (KPS) of ≥60 or Eastern Cooperative Oncology Group (ECOG) performance score of 0–2.

### Protocol Procedures

**Figure 1:**
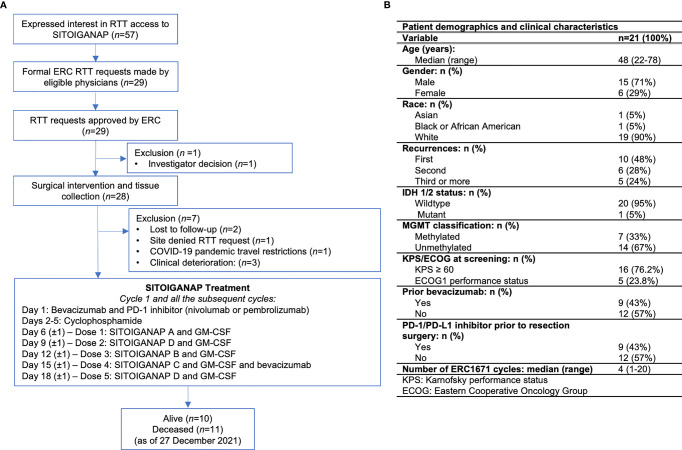
SITOIGANAP Right-to-Try program schema, and patient demographics. **(A)** ERC received 57 informal inquires on the RTT program. Twenty-nine formal requests for patient access to the SITOIGANAP RTT program were received and approved, and 21 rGBM patients completed at least one full cycle of SITOIGANAP. SITOIGANAP doses A–C are the allogeneic components. SITOGIANAP dose D is the autologous component. Cycle 1 starts on day 1 with bevacizumab and PD-1 inhibitor (nivolumab or pembrolizumab) administration, followed by four days of cyclophosphamide on days 2-5. SITOIGANAP A is a administered on day 6, SITOIGANAP D on day 9, SITOIGANAP B on day 12, SITOIGANAP C on day 15, and SITOIGANAP D on day 18. Each SITOIGANAP cycle is 28 days. The treatment with anti-programmed cell death protein-1 monoclonal antibodies (pembrolizumab and nivolumab) was administered at the standard dosing from other malignancies (pembrolizumab 200 mg every 3 weeks, nivolumab 480 mg every 4 weeks) and was started prior to surgical intervention (neoadjuvant) or on cycle 1, day 1. **(B)** Table of patient demographics and clinical characteristics.

SITOIGANAP was administered in combination with GM-CSF following low-dose cyclophosphamide to support immune system priming. Cyclophosphamide was administered a few days before each immunization cycle to deplete immune inhibitory cells in the patient (9). The SITOIGANAP vaccine was administered by intradermal injection. Co-administration of GM-CSF locally enhances the localized SITOIGANAP immune response. The composition of SITOIGANAP was described in detail elsewhere (9, 10). Briefly, one dose of SITOIGANAP (i.e., SITOIGANAP A through D, **Figure 1A**) consists of whole tumor cells (between 1 × 10^5^ and 1 × 10^6^ or tumor cell lysate (between 1 × 10^5^ and 1 × 10^6^. Before injection, 500 µg of GM-CSF (Leukine^®^) was added to each vaccine dose, and the combined volume was injected intradermally adjacent to the inguinal lymph nodes.

Each SITOIGANAP treatment cycle is 28 days long. Cyclophosphamide (Cytoxan^®^) was given orally (2 × 25 mg capsules per day) for 4 days (days 2–5) at the beginning of each cycle (**Figure 1A**). SITOIGANAP immunizations (A–D) were administered on days 6, 9, 12, 15, and 18. Patients received 5–10 mg/kg of bevacizumab (Avastin^®^) infusion on days 1 and 15 of each 28-day cycle. Anti-PD-1 monoclonal antibodies were administered at doses used for other malignancies (nivolumab 480 mg q4weeks and pembrolizumab 200 mg q3weeks) and were started at cycle 1, day 1. Nine patients were treated with neoadjuvant pembrolizumab or nivolumab per their treating neuro-oncologist’s choice. Treatment cycles were continued every 28 days until the progression of disease or intolerance.

Humoral immunologic response (CD3^+^/CD4^+^ T-lymphocyte count) was measured at baseline, at selected times during the vaccination, and at the time of disease progression or end of study (EOS). Patients underwent brain MRI as part of standard care before starting cycle 1 and every 8 weeks until disease progression and whenever progression was suspected on clinical symptoms. Tumor response was assessed using iRANO response criteria for high-grade gliomas by local physician self-report. Safety was evaluated throughout the trial by monitoring the incidence of AEs reported per agreement with participating physicians and prompted annually as a part of the annual review and reporting process between ERC and the DHHS.

### Outcomes

PFS was defined as the time from protocol entry to the date of reported progression or death due to any cause. Protocol entry was defined as the date of resection surgery for GBM tissue used to generate SITOIGANAP. OS was measured as the time from protocol entry until death. Immune monitoring in the peripheral blood (including but not limited to CD3^+^/CD4^+^ T-lymphocyte counts) was performed per local clinical standards and reported when prompted by ERC.

### Statistical Approach

General descriptive statistics include the number of observed values, mean, SD, median, and range. The number and percentage of subjects in each category were reported for categorical variables. Measures of OS and PFS were calculated by the Kaplan–Meier methods. Differences between survival curves were assessed by the log-rank test. Correlations between survival times and CD3^+^/CD4^+^ T-lymphocyte counts were by Spearman’s method. Survival times taken from the literature were averaged across studies by the harmonic mean, weighted by sample size. Values of percent surviving were averaged across studies weighted by sample size.

## Results

### SITOIGANAP Right-To-Try/Expanded Access Program Patient Characteristics

From June 2018 to December 2021, a total of 57 potential rGBM patients (or eligible physicians) contacted ERC and expressed interest in RTT access to SITOIGANAP (ERC1671/Gliovac™). As of December 27, 2021, 57 inquiries resulted in 29 formal applications, all of which were approved. Of the 29, one patient did not have surgery, six patients were not treated with SITOIGANAP, and one withdrew before completing one cycle of SITOIGANAP. Thus, this report was based on 21 rGBM patients who completed at least one cycle of SITOIGANAP therapy. A diagram depicting the flow of patients through the SITOIGANAP RTT request and enrollment process is provided in **Figure 1A**.

**Figure 1B** shows the personal and clinical characteristics of the study cohort (N = 21) as of protocol entry. The cohort is generally similar to rGBM patients in recent trials (4, 17, 18). The patients are predominantly male (71%) and range in age from 22 to 78, with 48% at least 50 years old. The *O* ^6^ -methylguanine-DNA methyltransferase promoter (*MGMT*) was methylated in 33% of patients (n = 7) and unmethylated in 67% (n = 14). One patient had isocitrate dehydrogenase (IDH)-mutant astrocytoma but was included in our rGBM study since enrollment occurred prior to cIMPACT-Now update 6 guidelines being published (19). Nine patients (43%) had failed to respond to earlier treatment with bevacizumab. Regarding PD-1 inhibitors and the debulking surgery, nine patients (43%) received nivolumab, three of them neoadjuvantly. The remaining 12 patients (57%) received pembrolizumab, six of them neoadjuvantly.

### Clinical Safety

SITOIGANAP was well tolerated, with no treatment-related serious AEs (SAE) reported. In accordance with the federal RTT law, SAEs were collected and reported to the Secretary of the DHHS (20). As of the analysis cutoff date, two patients experienced SAE unrelated to SITOIGANAP: one patient experienced encephalitis and weakness, and the other patient experienced fever, lethargy, and sepsis related to resection surgery. The administration of PD-1 inhibitors nivolumab or pembrolizumab was not associated with any unanticipated toxicities in the study cohort.

### Treatment Outcomes

**Figure 2:**
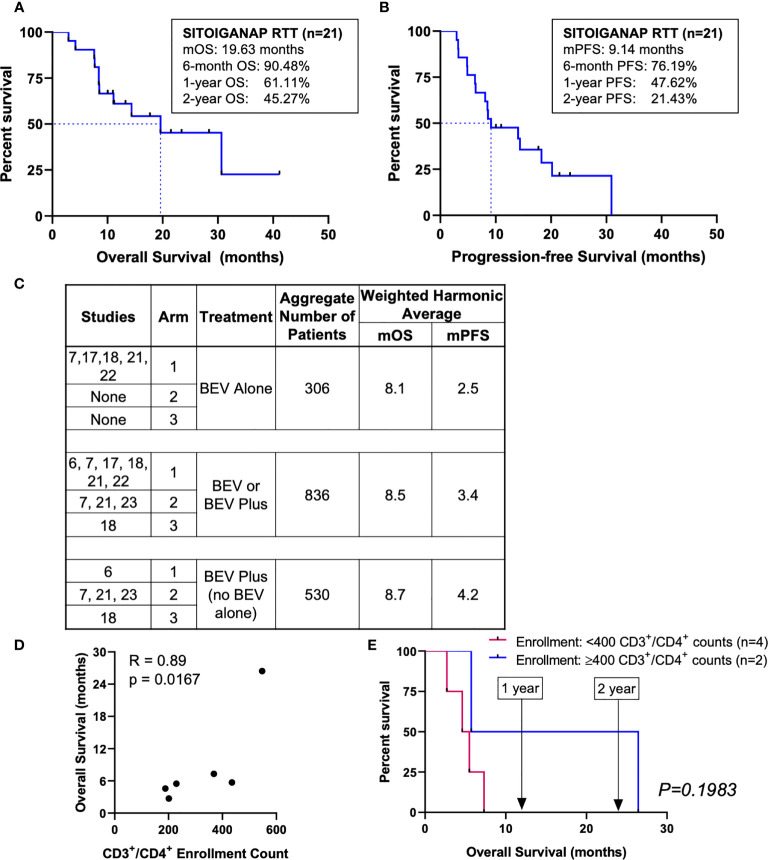
Overall survival, progression-free survival of SITOIGANAP RTT, comparisons with other reports, and CD3^+^/CD4^+^ T-lymphocyte counts correlate with overall survival. (A) Median OS was 19.63 months (n=21). (B) Median PFS was 9.14 months (C) Median OS and mPFS of SITOIGANAP + GM-CSF + cyclophosphamide+ bevacizumab + nivolumab/pembrolizumab is superior to previously published recurrent GBM studies using bevacizumab (BEV) alone or in combination. (See main text for detailed references). (D) Correlation between enrollment values of CD3^+^/CD4^+^ T-lymphocytes and overall survival (calculated from the day of the first SITOIGANAP cycle until death). (E) Kaplan-Meier survival analysis of enrollment CD3^+^/CD4^+^ T-lymphocyte counts.

At the time of analysis, 10 of the 21 patients (47.62%) were alive, with a mOS of 19.63 months (95% CI: 8.42–not estimable [na]). The 6-month, 1-year, and 2-year survival rates were 90.48%, 61.11%, and 45.27%, respectively (**Figure 2A**). The mPFS was 9.14 months (95% CI: 6.25–20.19), with 6-month, 1-year, and 2-year PFS rates of 76.19%, 47.62%, and 21.43%, respectively (**Figure 2B**). The mOS and mPFS of the SITOIGANAP regimen compare favorably to the outcomes reported for bevacizumab in the rGBM literature (**Figure 2C**) **(** 6, 7, 17, 18, 21–23).

### Clinical Efficacy: CD3^+^/CD4^+^ T-Cell Correlations With Overall Survival

CD3^+^/CD4^+^ helper T-lymphocyte counts (cells/mm^3^) were monitored in peripheral blood at enrollment (baseline) and every 2 weeks during the SITOIGANAP treatment period. The CD3^+^/CD4^+^ T-lymphocyte counts at enrollment (**Figure 2D**) and End-of-Study (data not shown) both correlated with OS measured from the first SITOIGANAP cycle (r = 0.89, p = 0.0167; r = 0.87, p = 0.0167, respectively). Only CD3^+^/CD4^+^ counts from patients whose peripheral blood samples were collected at both time points (n = 6) were included in the analysis. Of note, two of the six patients (33%) had CD3^+^/CD4^+^ ≥400 cells/mm^3^ at enrollment, with a mOS of 16.08 months (95% CI: 5.72–na) and 2-year survival rate of 50% (**Figure 2E**). Patients with CD3^+^/CD4^+^ <400 cells/mm^3^ at enrollment (67%) had a mOS of 5.05 months (95% CI: 2.70–na; p = 0.1983, log-rank test).

Although the data are sparse and not statistically significant, patients who received neoadjuvant anti-PD-1 had both somewhat higher mean CD3^+^/CD4^+^ counts at enrollment and longer median survival, compared to those who received adjuvant anti-PD-1 (neoadjuvant N = 2, mean CD3^+^/CD4^+^ = 374 cells/mm^3^, mOS = 14.57 [95% CI: 2.70–na]; adjuvant N = 4, mean CD3^+^/CD4^+^ = 305 cells/mm^3^, mOS = 5.61 [95% CI: 4.60–na], both comparisons not significant).

### The Impact of Neoadjuvant Anti-PD-1 Inhibitor Administration Prior to Resection Surgery on Overall Survival and Progression-Free Survival in Patients Receiving SITOIGANAP Needs to Be Further Explored

As of the analysis cutoff date, there have been four deaths in patients receiving neoadjuvant anti-PD-1 antibody and seven in patients receiving adjuvant anti-PD-1 antibody only. Patients who received PD-1 inhibitor nivolumab or pembrolizumab prior to resection surgery demonstrated a mOS of 30.64 months (95% CI: 2.93–na), 6-month, 1-year, and 2-year survival rates of 77.78%, 66.67%, and 66.67%, respectively, as compared to the adjuvant group (p = 0.3165, log-rank test; **Supplementary Figure 1A**). Patients in the adjuvant-only group had a mOS of 14.37 months (95% CI: 8.42–na), 6-month, 1-year, and 2-year survival rates of 100%, 55.56%, and 18.52%, respectively. Median PFS was 8.83 months (95% CI: 4.83–14.37) in the adjuvant group and 18.25 months (95% CI: 2.93–na) in the neoadjuvant group (p = 0.3841, log-rank test; **Supplementary Figure 1B**). Though numerically the mOS in the neoadjuvant PD-1 inhibitor group was much higher than in the adjuvant-only group (30.64 vs. 14.37 months), the difference was not statistically significant and needs to be validated with a larger number of patients in a randomized design.

### MGMT Status

In patients with methylated MGMT (n = 7), mOS was 30.64 months (95% CI: 7.66–na) from protocol entry, with 6-month, 1-year, and 2-year survival rates of 100%, 57.14%, and 57.14%, respectively. Although the mOS of patients with methylated MGMT is the same as the mOS of patients who received neoadjuvant anti-PD-1 (i.e., mOS = 30.64 months), two patients with methylated MGMT received neoadjuvant anti-PD-1 antibody, and the other five received adjuvant anti-PD-1. In patients with unmethylated MGMT (n = 14), mOS was 19.63 months (95% CI: 7.56–na) from protocol entry, with 6-month, 1-year, and 2-year survival rates of 85.71%, 62.5%, and 39.06%, respectively (p = 0.9275, log-rank test,**Supplementary Figure 1C**).

### Long-Time Responders to Immunotherapy

Beneficial effects of immune therapies are often observed at later time points following several cycles (24). Among patients who received at least six SITOIGANAP cycles (n = 10), mOS was 30.64 months (95% CI: 11.11–na), with 1-, 2-, and 3-year survival rates of 90%, 57.86%, and 28.93%, respectively. In contrast, mOS was 8.42 months (95% CI: 4.18–na) among patients who received fewer than six SITOIGANAP cycles (n = 11), with 1- and 2-year survival rates of 36.36% and 36.36%, respectively (p = 0.0323, log-rank test, **Supplementary Figure 1D**).

The long-time responders were equally comprised of patients who received neoadjuvant (n = 5) and adjuvant (n = 5) anti-PD-1 therapy.

## Discussion

The authors believe this is the first report of results from an RTT program for rGBM in the United States. A large proportion of the patients interested in SITOIGANAP qualified for the treatment under RTT (or similar programs internationally), with 50% of requests for access leading to protocol entry (surgery with tissue collection, 28/56 patients). Enrolled patients potentially benefited from the SITOIGANAP regimen without undue risk.

Overall, the mOS of 19.63 months and mPFS of 9.14 months (from resection surgery) compare favorably with the average mOS of 7.8 months and mPFS of 2.4 months derived from the literature on trials in rGBM (**Supplementary Figure 2A** [weighted harmonic means]) (4, 6, 7, 17, 18, 21–23, 25–28). Data derived from three additional representative, contemporary studies with bevacizumab (29), BCNU (30), and lomustine (31) all yielded estimated survival functions that are statistically inferior (log-rank p < 0.0001) to that demonstrated here for the SITOIGANAP regimen (**Supplementary Figure 2B–D**). Moreover, none of the combined totals of 112 patients in these trials (29–31) survived 24 months. In contrast, 45% of the 21 patients on the SITOIGANAP regimen survived at least 24 months (**Figure 2A**). A similar signal of the benefit of the SITOIGANAP regimen was obtained when the mOS of the SITOIGANAP cohort was compared to that reported for bevacizumab in recent trials in rGBM (**Figure 2C**) **(** 6, 7, 17, 18, 21–23). Equally important, we observed no new, reportable safety concerns associated with the SITOIGANAP regimen.

This study also suggests that the CD3^+^/CD4^+^ T-lymphocyte counts at the start of treatment may improve patient selection in future studies of SITOIGANAP. This study was exploratory and subject to the usual limitations of single-arm designs with self-selected participants. However, these results encourage the development of a well-controlled, phase III design.

## Data Availability Statement

The raw data supporting the conclusions of this article will be made available by the authors, without undue reservation.

## Ethics Statement

The studies involving human participants were reviewed and approved by the University of California Irvine IRB. The patients/participants provided their written informed consent to participate in this study.

## Author Contributions

The following authors have contributed significantly: DAB, TT, and AS to the experimental design; DAB, X-TK, and BF to its implementation; and all authors to the analysis and interpretation of the data. DAB, TT, and NL wrote the first draft of the manuscript. All authors contributed to manuscript revision and read and approved the submitted version.

## Funding

This study was supported by funding from Epitopoietic Research Corporation (ERC). The study is an investigator-initiated study (DAB) funded by ERC. DAB designed the study in collaboration with the study sponsor and the other investigators and managed the clinical trial database and performed the statistical analysis. The corresponding author had full access to all the data in the study and had final responsibility for the decision to submit it for publication. Grant number: UCI Cancer Center Award [P30CA062203] from the National Cancer Institute. DAB has received research support from ERC.

## Conflict of Interest

The following authors are on the ERC advisory board or board of directors, employed by ERC, or own ERC shares: TC, VS, JE, and AS.

The remaining authors declare that the research was conducted in the absence of any commercial or financial relationships that could be construed as a potential conflict of interest.

## Publisher’s Note

All claims expressed in this article are solely those of the authors and do not necessarily represent those of their affiliated organizations, or those of the publisher, the editors and the reviewers. Any product that may be evaluated in this article, or claim that may be made by its manufacturer, is not guaranteed or endorsed by the publisher.
