# Supplementary material for: A Prospective, Cohort Study of SITOIGANAP to Treat Glioblastoma When Given in Combination With Granulocyte-Macrophage Colony-Stimulating Factor/Cyclophosphamide/Bevacizumab/Nivolumab or Granulocyte-Macrophage Colony-Stimulating Factor/Cyclophosphamide/Bevacizumab/Pembrolizumab in Patients Who Failed Prior Treatment With Surgical Resection, Radiation, and Temozolomide
Source: Front Oncol. 2022 Jun 28;12:934638. doi: 10.3389/fonc.2022.934638 (PMC9273968; doi:10.3389/fonc.2022.934638)
Supplement: Supplementary file 1 [file DataSheet_1.docx]

Methods to Supplementary Figure 2B,C,D (comparative Kaplan-Meier curves)

In an effort to compare the survival data obtained in our study to other studies available from the published literature, we proceeded as follows. Because the original patient data files from other studies were not accessible to us, we relied on the published details, such as OS-6, OS-12, mOS and Kaplan-Meier survival graphs. The published Kaplan-Meier graphs were maximally enlarged and then overlaid with a purposefully created fine grid on a high-resolution 27-inch computer monitor. This enabled us to read each data point (i.e., days of survival) for each patient. Using the data collected in this manner, we re-created the respective Kaplan-Meier graph and used this copy to compare to the data of our study, inclusive of the calculation of p-values. While we acknowledge that creating such a replicate might not be 100% precise, we do consider our meticulous procedure as a sufficiently reliable approach to allow reasonable comparisons.
