# Supplementary figures and images for: A Prospective, Cohort Study of SITOIGANAP to Treat Glioblastoma When Given in Combination With Granulocyte-Macrophage Colony-Stimulating Factor/Cyclophosphamide/Bevacizumab/Nivolumab or Granulocyte-Macrophage Colony-Stimulating Factor/Cyclophosphamide/Bevacizumab/Pembrolizumab in Patients Who Failed Prior Treatment With Surgical Resection, Radiation, and Temozolomide
Source: Front Oncol. 2022 Jun 28;12:934638. doi: 10.3389/fonc.2022.934638 (PMC9273968; doi:10.3389/fonc.2022.934638)

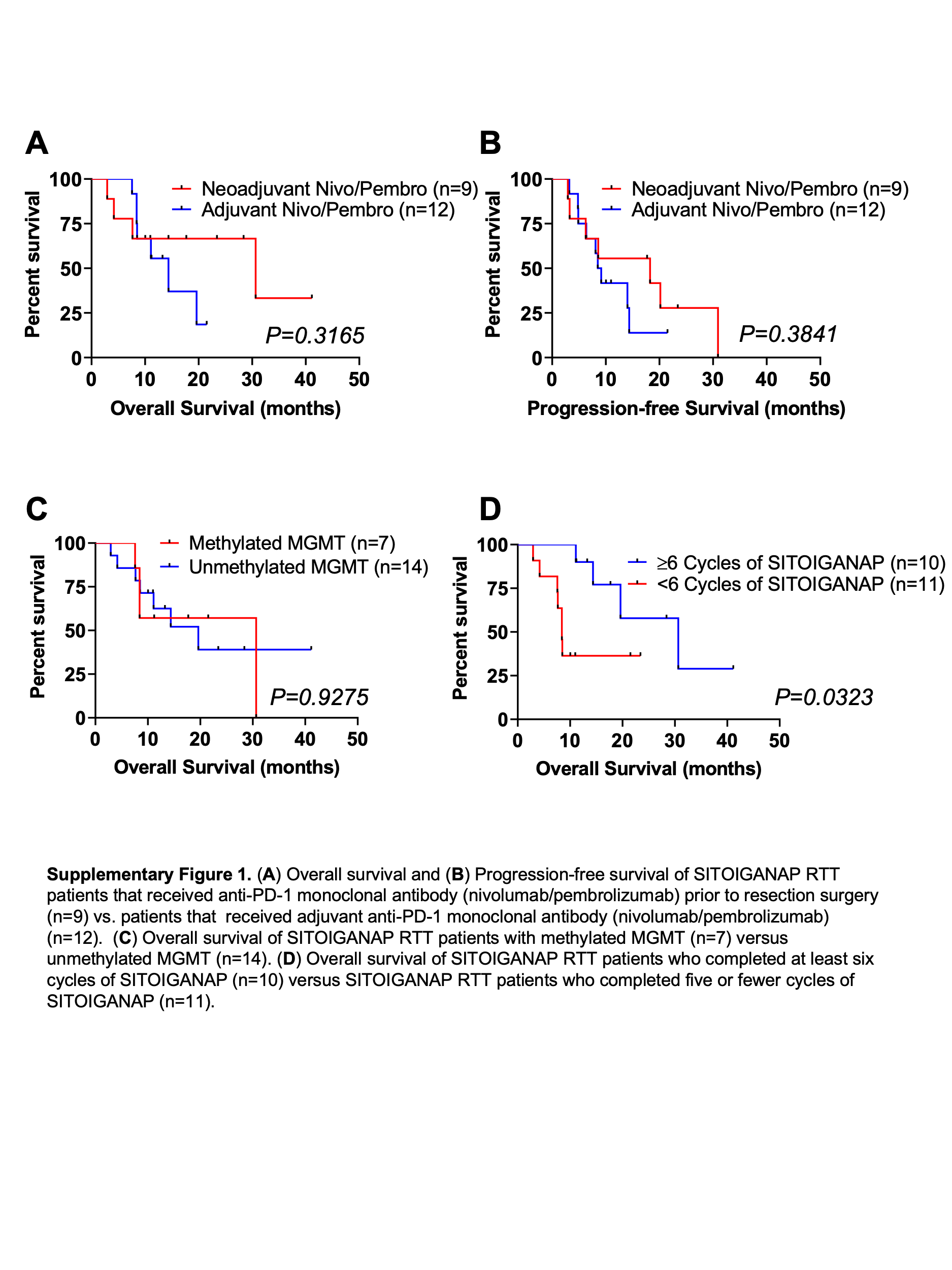

Supplement: Supplementary file 2 [file Image_1.tiff]

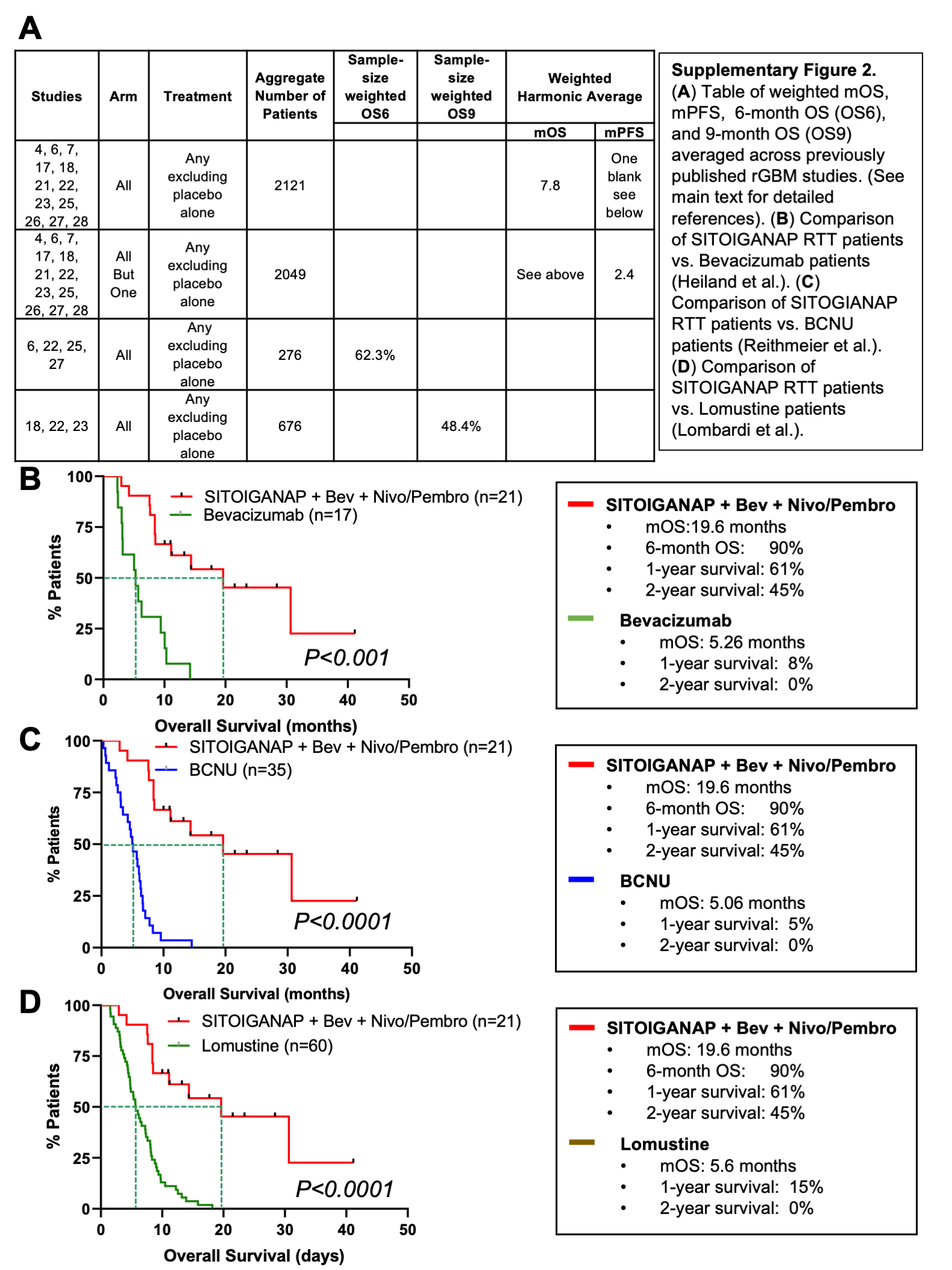

Supplement: Supplementary file 3 [file Image_2.tiff]
